# Retrospective study of 115 patients with diabetes mellitus complicated by retrograde ejaculation

**DOI:** 10.1186/s12610-026-00303-7

**Published:** 2026-02-23

**Authors:** Chuangui Li, Huan Wang, Siqi Li, Tianzi Zhang, Hao Li, Chongbo Wang

**Affiliations:** 1 Department of Urology III, Baoding First Central Hospital, Baoding, Hebei Province China; 2 Faculty of Humanities and Social Sciences, City University of Macau, Macau, China; 3 Graduate School, Hebei Medical University, Shijiazhuang, Hebei Province China

**Keywords:** Diabète sucré, Ejaculation rétrograde, Durée de la Maladie, Plaisir sexuel, Code de Classification de la Fertilité R698 Code du document A

## Abstract

**Background:**

Retrograde ejaculation is a significant complication of diabetes mellitus, yet its association with other sexual dysfunctions remains under-characterized. Current literature often treats it as an isolated fertility issue, overlooking potential co-morbidities. This study aimed to comprehensively profile the clinical characteristics of diabetic men with retrograde ejaculation and investigate the independent risk factors for severe sexual dysfunction, specifically moderate-to-severe erectile dysfunction.

**Results:**

A retrospective analysis of 115 diabetic patients (mean age 39.9 years) confirmed retrograde ejaculation revealed a high prevalence of “triple dysfunction”: 80.0% had complete retrograde ejaculation, 54.8% suffered from moderate-to-severe erectile dysfunction, and 39.1% reported anorgasmia. Type 1 diabetic patients, despite being younger and having higher testosterone levels than Type 2 patients, exhibited worse functional outcomes, including a significantly higher rate of anorgasmia (65.5% vs. 30.2%). Multivariate logistic regression identified diabetes duration (OR 1.70, *P* < 0.001), age (OR 1.23, *P* < 0.001), and glycated hemoglobin (OR 1.70, *P* = 0.004) as independent predictors of moderate-to-severe erectile dysfunction. Lifestyle factors such as smoking and alcohol consumption were not significant independent predictors in the adjusted model.

**Conclusions:**

Retrograde ejaculation in diabetic men is rarely an isolated symptom but part of a complex syndrome frequently co-existing with severe erectile dysfunction and anorgasmia. The severity of this dysfunction is primarily driven by the chronicity of diabetes and long-term glycemic instability rather than age or lifestyle factors alone. Clinicians should adopt a holistic approach, screening for co-morbid sexual and psychological dysfunctions to guide precise management.

## Résumé

**Contexte:**

Les patients diabétiques atteints d’éjaculation rétrograde (ER) rencontrent souvent des défis sexuels et reproductifs multiples ; mais les relations entre le type de diabète, l’évolution de la maladie, le contrôle métabolique, la fonction érectile, l’historique de fertilité et le plaisir sexuel restent insuffisamment caractérisés. Cette étude visait à analyser les liens entre l’âge, les antécédents de fertilité, le plaisir sexuel, la durée du diabète, les niveaux d’hémoglobine A1c (HbA1c) et la dureté érectile chez les patients diabétiques diagnostiqués avec une ER.

**Résultats:**

Parmi 115 patients diabétiques atteints d’ER, 60,9 % ont déclaré ressentir du plaisir sexuel, tandis que 80 % présentaient une dysfonction érectile. Comparés aux patients diabétiques de type 2 (*n*=87), ceux atteints de diabète de type 1 (*n*=28) étaient significativement plus jeunes (34,1±7,8 contre 41,7±7,6 ans, *P*<0,01), avaient une durée de diabète plus longue (*P*<0,01) et présentaient des taux plus élevés d’anorgasmie (64,3 % versus 31 %, *P*=0,004) et d’absence d’enfants (46,4 % versus 13,8 %, *P*<0,01). Le Score de Dureté Erectile (SDE) était significativement plus faible chez les patients qui ont rapporté n’avoir aucun plaisir sexuel (*P*<0,01). Les patients infertiles étaient plus jeunes (*P*<0,01) et présentaient un SDE plus faible (*P*=0,035). L’analyse des courbes ROC a indiqué qu’un SDE plus faible était fortement corrélé à l’anorgasmie (aire sous la courbe : AUC =0,831).

**Conclusion:**

Chez les patients diabétiques qui présentent une éjaculation rétrograde, le plaisir sexuel, les antécédents de fertilité et la dureté érectile sont étroitement liés au type de diabète, à la durée de la maladie et à l’âge. Les patients atteints de diabète de type 1 consultent à un plus jeune âge, et présentent un risque plus élevé de dysfonction érectile, d’anorgasmie et d’absence d’enfants. Ces résultats soulignent l’importance d’une évaluation et d’une prise en charge individualisées dans cette population, avec une attention particulière aux types de diabète et à l’évolution de la maladie associée.

## Introduction

Diabetes mellitus (DM) is a chronic metabolic disorder characterized by persistent hyperglycemia resulting from defects in insulin secretion, insulin action, or both. The global burden of DM has escalated alarmingly; according to the latest *IDF Diabetes Atlas*, the prevalence of diabetes affects approximately 537 million adults worldwide, showing a concerning trend toward younger onset [1].

Among the myriad complications of diabetes, sexual dysfunction is highly prevalent but often under-addressed [2]. While erectile dysfunction (ED) is widely recognized, ejaculatory dysfunction (EjD) affects a substantial proportion of male diabetic patients. Retrograde ejaculation (RE) is a specific form of EjD characterized by the propulsion of semen into the urinary bladder during orgasm due to the failure of the bladder neck to close. This condition is primarily attributed to diabetic autonomic neuropathy [3] and is often associated with other vascular or neurological deficits. Recent studies published in *Basic and Clinical Andrology* have highlighted the complex genetic and molecular mechanisms linking diabetes to sexual dysfunction, such as the causal role of specific signaling pathways in ED [4, 5].

Although RE accounts for less than 2% of male infertility cases in the general population, its management remains a significant clinical challenge. Notably, in patients initially diagnosed with azoospermia, the prevalence of RE can be significantly higher due to misdiagnosis. As demonstrated by Perrin et al. in this journal, while RE is a known cause of infertility, optimizing sperm retrieval methods from post-ejaculatory urine can successfully restore fertility potential, underscoring the importance of accurate diagnosis [6, 7].

However, current literature often treats RE as an isolated fertility issue. Emerging evidence suggests that in diabetic men, RE is rarely an isolated symptom but frequently co-exists with severe erectile and orgasmic dysfunction, forming a complex “triple dysfunction” syndrome that severely compromises quality of life [8, 9]. Currently, there is a paucity of clinical studies comprehensively profiling the interplay between metabolic control, disease duration, and this composite sexual dysfunction in the diabetic population. Therefore, this retrospective study aimed to analyze the clinical characteristics of 115 diabetic patients with RE, specifically investigating the independent risk factors for severe dysfunction to guide precise clinical management.

## Patients and methods

### Study design and participants

This retrospective study included 115 male patients diagnosed with DM complicated by RE who visited the Department of Andrology at Baoding First Central Hospital from January 2020 to June 2025. The study protocol was approved by the Ethics Committee of Baoding First Central Hospital (Approval No.: Kuai [2024] 170) and conducted in accordance with the Declaration of Helsinki. Informed consent was obtained from all participants.

### Inclusion and exclusion criteria

#### Inclusion criteria

(1) Male aged 18–60 years; (2) Confirmed history of Type 1 (T1DM) or Type 2 Diabetes Mellitus (T2DM); (3) Comparison of clinical symptoms and urinalysis confirming the diagnosis of RE; (4) Married or in a stable relationship with regular sexual activity.

#### Exclusion criteria

**Fig. 1:**
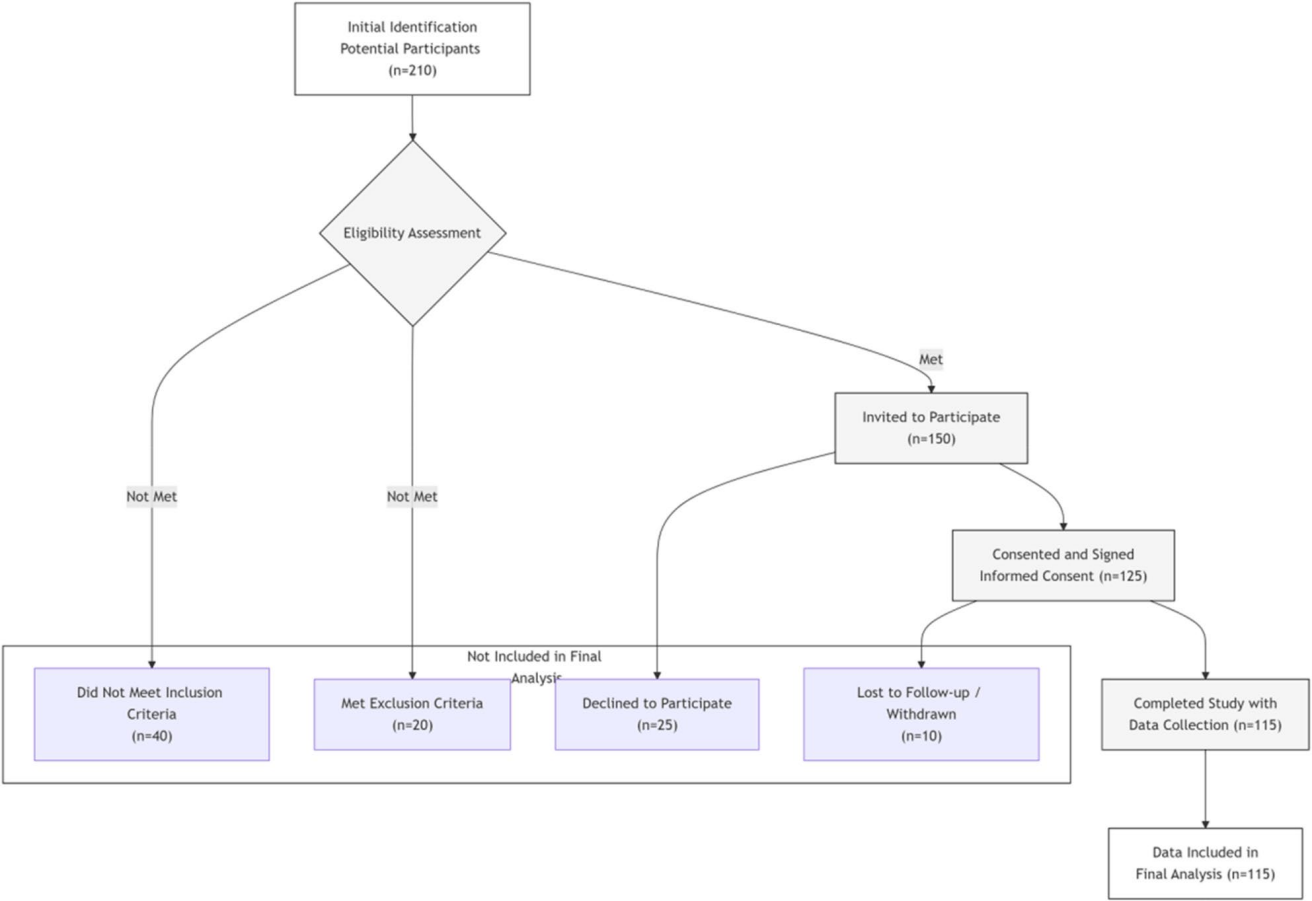
Flowchart of study participant screening and inclusion. This figure illustrates the complete process from the initial identification of 210 potential patients, through eligibility assessment, exclusion of those who did not meet the criteria, obtaining informed consent, to the final inclusion of 115 patients who completed data collection and analysis

(1) History of pelvic, urethral, or prostate surgery (e.g., TURP) that could mechanically cause RE; (2) Use of medications known to affect ejaculation (e.g., alpha-blockers, antidepressants); (3) Severe genitourinary malformations; (4) Clinically significant psychiatric disorders or untreated severe systemic diseases.The screening process is illustrated in Fig. 1.

### Data collection and clinical assessment

#### Demographic and metabolic profiling

Baseline data were retrieved from electronic medical records, including age, Body Mass Index (BMI), DM type, and lifestyle factors (smoking history and alcohol consumption). Detailed diabetes history was recorded, specifically the disease duration. Glycemic control was assessed by measuring glycosylated hemoglobin (HbA1c) levels using venous blood samples (reference range: 4%–6%).

#### Semen analysis and diagnosis of RE

Patients abstained from sexual activity for 2–7 days prior to sample collection. Semen analysis was performed using computer-aided sperm analysis (CASA) in strict accordance with the World Health Organization (WHO) Laboratory Manual for the Examination and Processing of Human Semen (6th Edition, 2021). Sperm morphology was assessed using WHO criteria. The definitions for semen parameters were established as follows based on the WHO reference limits (5th centile): Oligozoospermia (sperm concentration < 16 × 10⁶/ml), Asthenozoospermia (progressive motility < 30%), and Teratozoospermia (normal forms < 4%).

The diagnosis of RE was confirmed based on the following protocol: Patients were instructed to urinate and empty their bladder before masturbation. Post-ejaculation urine was immediately collected and centrifuged. The presence of sperm (> 10/High Power Field [HPF]) or a positive fructose test in the post-ejaculatory urine confirmed the diagnosis. Patients were subsequently classified into Complete RE (zero antegrade semen volume) or Partial RE (presence of some antegrade semen) [10].

Quality control (QC) was strictly maintained throughout the study period. Daily internal quality control (IQC) was performed using standard commercial latex beads to calibrate sperm concentration. Furthermore, the laboratory participates in an External Quality Assessment (EQA) scheme organized by the National Center for Clinical Laboratories to ensure the accuracy and reproducibility of semen parameters.

#### Assessment of sexual function and psychological status

#####  Erectile function

Assessed using the Erection Hardness Score (EHS). Patients were categorized into two groups for analysis: Mild/Normal function (EHS 3–4) and Moderate-to-Severe ED (EHS 1–2), defined as the inability to achieve vaginal penetration [11].

##### Orgasmic function

Presence or absence of orgasm (anorgasmia) was self-reported.

##### Psychological status

Anxiety and depression symptoms were screened using standard clinical interviews or validated questionnaires, recorded as binary outcomes (Yes/No).

### Statistical analysis

Data analysis was performed using SPSS version 29.0 (IBM Corp., Armonk, NY, USA) and R software. Continuous variables were tested for normality using the Shapiro-Wilk test and presented as Mean ± Standard Deviation (SD). Categorical variables were expressed as frequencies and percentages (n, %). Group comparisons (e.g., T1DM vs. T2DM, Short vs. Long Duration) were conducted using the independent sample t-test for continuous variables and the Chi-square test or Fisher’s exact test for categorical variables. Correlations between disease duration and EHS were analyzed using Pearson’s correlation coefficient. To identify independent predictors of moderate-to-severe ED, univariate and multivariate logistic regression analyses were performed, presenting results as Odds Ratios (OR) with 95% Confidence Intervals (CI). A two-sided *P*-value < 0.05 was considered statistically significant.

## Results

### Baseline clinical characteristics and lifestyle profile

A total of 115 diabetic patients with confirmed RE were included in this retrospective analysis. The detailed demographic and clinical characteristics of the study cohort are summarized in Table 1.

**Table 1:** Baseline Clinical, Metabolic, and lifestyle characteristics of 115 patients

Characteristic	Value (*N* = 115)
Demographics	
Age (years)	39.88 ± 8.33
BMI (kg/m²)	26.13 ± 3.82
Diabetes Profile	
Duration of Diabetes (years)	8.49 ± 4.65
HbA1c (%)	9.80 ± 1.98
Type 1 Diabetes, n (%)	29 (25.2%)
Type 2 Diabetes, n (%)	86 (74.8%)
Lifestyle & Psychological Profile	
Smoking History, n (%)	49 (42.6%)
Alcohol Consumption, n (%)	41 (35.7%)
Anxiety/Depression, n (%)	45 (39.1%)
Sexual & Reproductive Function	
Testosterone (nmol/L)	19.12 ± 3.61
EHS Score (Mean ± SD)	2.42 ± 1.06
Moderate-to-Severe ED (EHS ≤ 2), n (%)	63 (54.8%)
Orgasm Present, n (%)	70 (60.9%)
Fertility History (Secondary Infertility), n (%)	90 (78.3%)
Semen Analysis Parameters	
Complete Retrograde Ejaculation^a^, n (%)	92 (80.0%)
Semen Volume (mL)	0.14 ± 0.35
Sperm Concentration (×10⁶/mL)	0.47 ± 1.13

Data are presented as mean ± standard deviation (SD) for continuous variables and number (percentage) for categorical variables

*Abbreviations*: *BMI* Body Mass Index, *HbA1c* Glycated Hemoglobin, *ED* Erectile Dysfunction, *EHS* Erection Hardness Score

^a^Complete Retrograde Ejaculation is defined as zero antegrade semen volume. Semen Analysis Parameters (Volume and Concentration) represent the mean values for the entire cohort (*n* = 115), including subjects with complete retrograde ejaculation (recorded as 0 mL volume and 0 concentration)

**Fig. 2:**
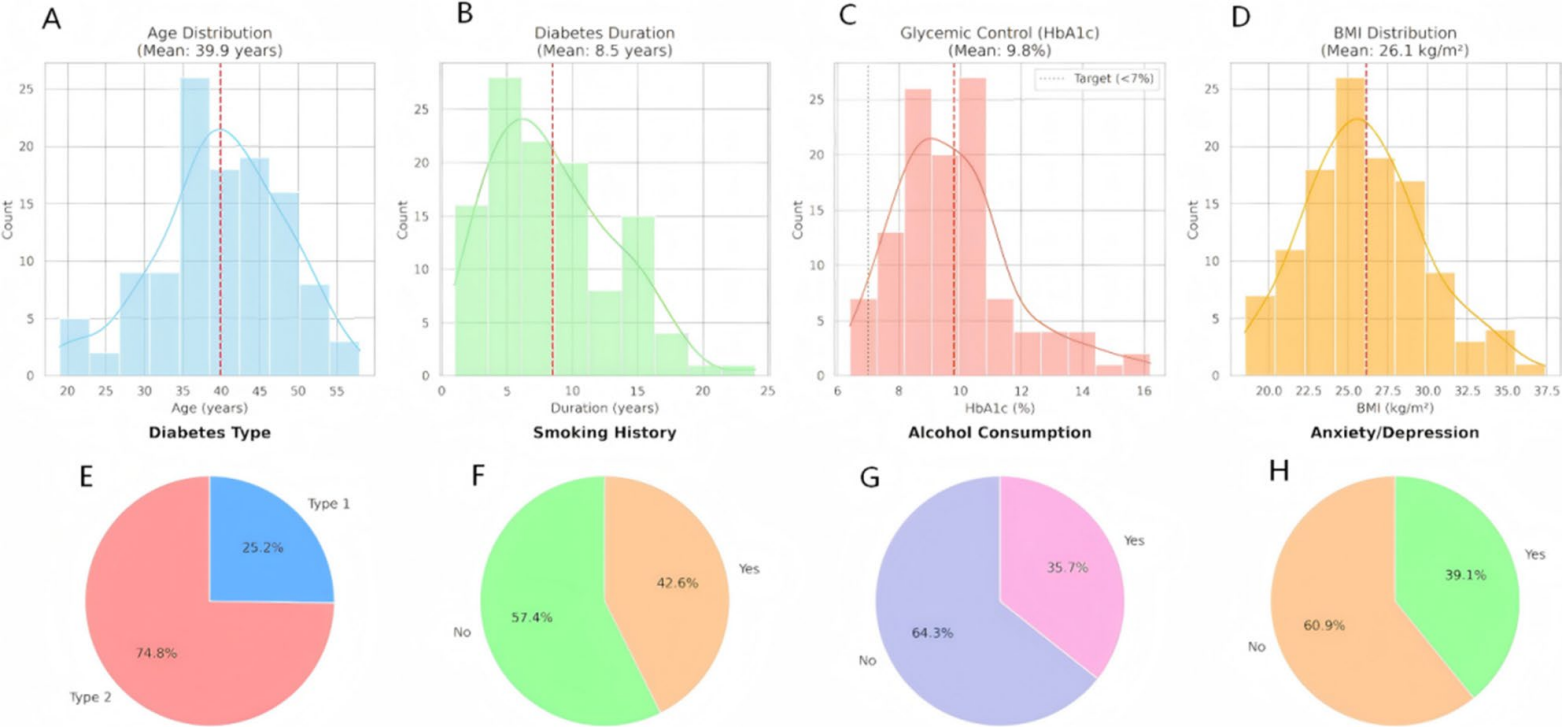
Comprehensive Baseline Clinical, Metabolic, and Lifestyle Profile of the Study Cohort. Visual representation of the demographic and clinical landscape of the 115 diabetic patients with retrograde ejaculation. **A-D **Distributions of Continuous Clinical Variables: Histograms with kernel density estimation (KDE) curves showing the distribution of (**A**) Age, (**B**) Duration of Diabetes, (**C**) Glycated Hemoglobin (HbA1c), and (**D**) Body Mass Index (BMI). The vertical red dashed lines indicate the mean value for the entire cohort. In panel (**C**), the vertical green dotted line represents the standard clinical target for glycemic control (< 7.0%). **E**-**H** Prevalence of Categorical Characteristics: Charts illustrating the proportional composition of the cohort regarding (**E**) Diabetes Type (Type 1 vs. Type 2), (**F**) Smoking History, (**G**) Alcohol Consumption, and (**H**) Psychological Status (Anxiety/Depression). *Abbreviations: BMI*,* Body Mass Index; HbA1c*,* Glycated Hemoglobin*

The study population was relatively young, with a mean age of 39.88 ± 8.33 years. The majority of patients were diagnosed with T2DM (74.8%), while 25.2% had T1DM. Despite the relatively young age, the cohort exhibited a substantial disease burden. The mean duration of diabetes was 8.49 ± 4.65 years, and glycemic control was generally suboptimal, with a mean HbA1c level of 9.80 ± 1.98%, as illustrated in the distribution plots in Fig. 2. The mean BMI was 26.13 ± 3.82 kg/m², indicating that the average patient was slightly overweight.

Regarding lifestyle and psychological factors, the prevalence of potential risk behaviors was notable. As shown in Fig. 2, 42.6% of patients had a history of smoking, and 35.7% reported regular alcohol consumption. Furthermore, psychological comorbidities were common, with 39.1% of the participants screening positive for anxiety or depression.

In terms of reproductive and sexual function, 80.0% (92/115) of patients presented with complete RE (zero antegrade semen volume). Although the mean serum testosterone level was within the normal physiological range (19.12 ± 3.61 nmol/L), erectile function was significantly impaired. The mean EHS was 2.42 ± 1.06, and more than half of the cohort (54.8%) suffered from moderate-to-severe ED (EHS ≤ 2).

### Severity of sexual and reproductive dysfunction

The spectrum of sexual dysfunction in this cohort extended beyond RE, presenting as a complex interplay of erectile, ejaculatory, and orgasmic impairments. The detailed distribution of these dysfunctions is presented in Table 2.

**Table 2:** Detailed profile of sexual dysfunction

Category	Count (*n* = 115)	Percentage
Erectile Function (EHS Grade)		
Grade 1 (Severe: Penis is larger but not hard)	27	23.50%
Grade 2 (Moderate: Hard but not hard enough for penetration)	36	31.30%
Grade 3 (Mild: Hard enough for penetration but not completely hard)	29	25.20%
Grade 4 (Normal: Completely hard and fully rigid)	23	20.00%
Ejaculatory Function		
Complete RE (0 mL antegrade semen)	92	80.00%
Partial RE (> 0 mL antegrade semen)	23	20.00%
Orgasmic Function		
Anorgasmia (No Orgasm)	45	39.10%
Orgasm Preserved	70	60.90%

Erection Hardness Score (EHS) is graded from 1 to 4. Moderate-to-Severe Erectile Dysfunction (ED) corresponds to EHS grades 1 and 2, indicating an inability to achieve penetration. Anorgasmia refers to the self-reported absence of orgasm during sexual activity

While all patients were diagnosed with RE, the severity varied. The majority (80.0%, *n* = 92) exhibited complete retrograde ejaculation, characterized by the total absence of antegrade semen volume, whereas only 20.0% retained partial antegrade emission.

Notably, erectile dysfunction (ED) was a highly prevalent comorbidity. As shown in Table 2, only 20.0% of patients maintained normal erectile function (EHS 4). The remaining 80.0% suffered from varying degrees of ED, with more than half of the cohort (54.8%) classified as having moderate-to-severe ED (EHS 1–2). This suggests that neurovascular impairment in these patients is widespread, affecting both the ejaculatory and erectile machinery.

Furthermore, sensory impairment was evident, as 39.1% of patients reported total anorgasmia (absence of orgasm), despite the presence of ejaculation (retrograde). Importantly, these functional deficits occurred in the context of a eugonadal state, as the mean serum testosterone level was 19.1 ± 3.6 nmol/L, effectively ruling out hypogonadism as a primary etiology for the observed dysfunction.

**Fig. 3:**
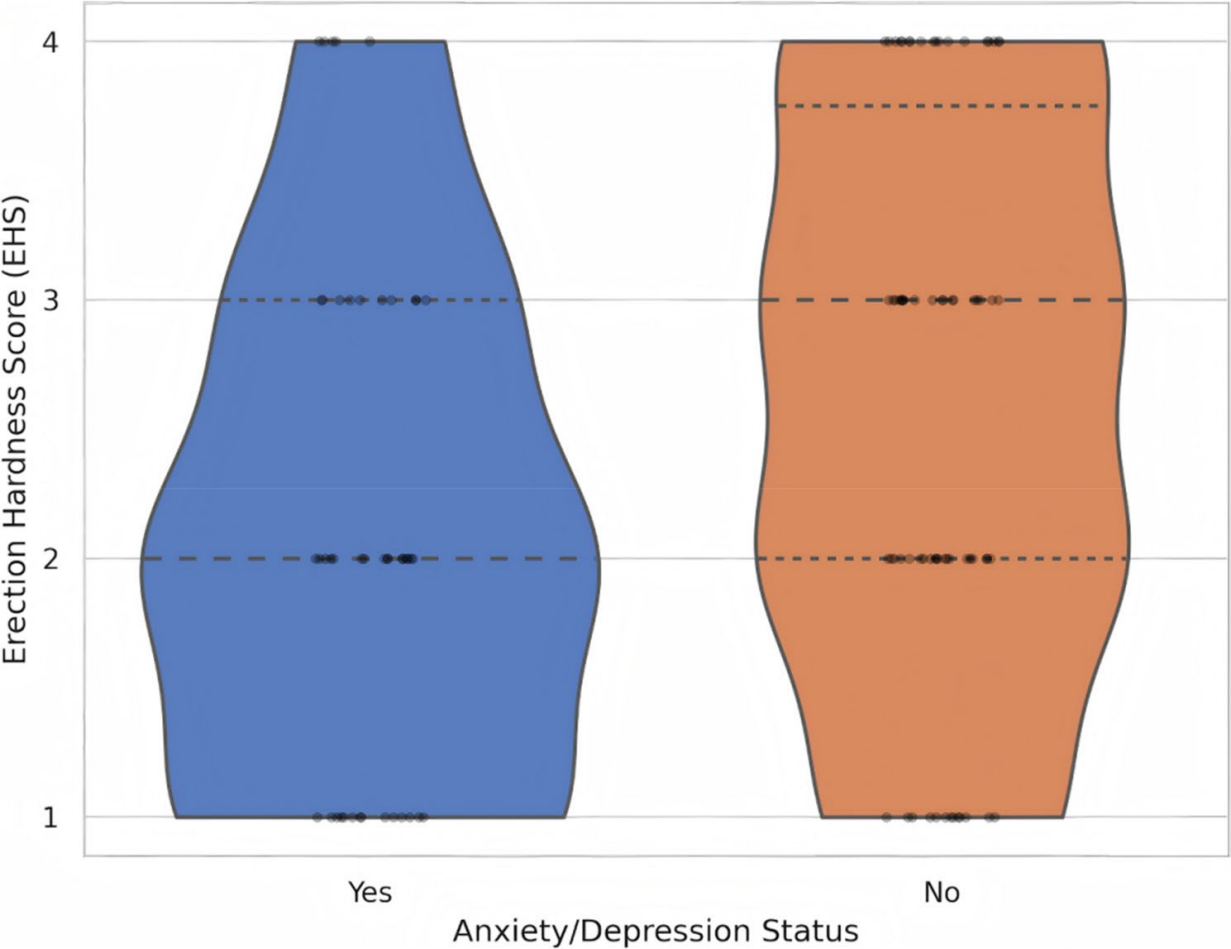
Impact of Psychological Burden on the Severity of Erectile Dysfunction. A violin plot illustrating the distribution density of Erection Hardness Scores (EHS) stratified by psychological status (Anxiety/Depression). The width of the violin shape at each EHS level (1–4) corresponds to the frequency of patients in that category. The plot reveals a distinct shift in distribution: patients with co-existing anxiety or depression (Right, Orange) show a higher density of scores concentrated at EHS 1 and 2 (Moderate-to-Severe ED), whereas those without psychological distress (Left, Blue) exhibit a more distributed profile with preserved erectile function (EHS 3 and 4). Individual data points (black dots) are overlaid to depict the raw data spread. *Abbreviations: EHS*,* Erection Hardness Score; ED*,* Erectile Dysfunction*

The impact of psychological factors on this “triple dysfunction” (RE, ED, and Anorgasmia) is visualized in Fig. 3. Patients with co-existing anxiety or depression demonstrated a visibly lower distribution of EHS scores compared to those without psychological distress. The violin plot reveals a density shift towards EHS 1–2 in the anxiety group, highlighting the exacerbating role of psychological burden in this complex clinical picture.

### Impact of diabetes duration on erectile function

**Fig. 4:**
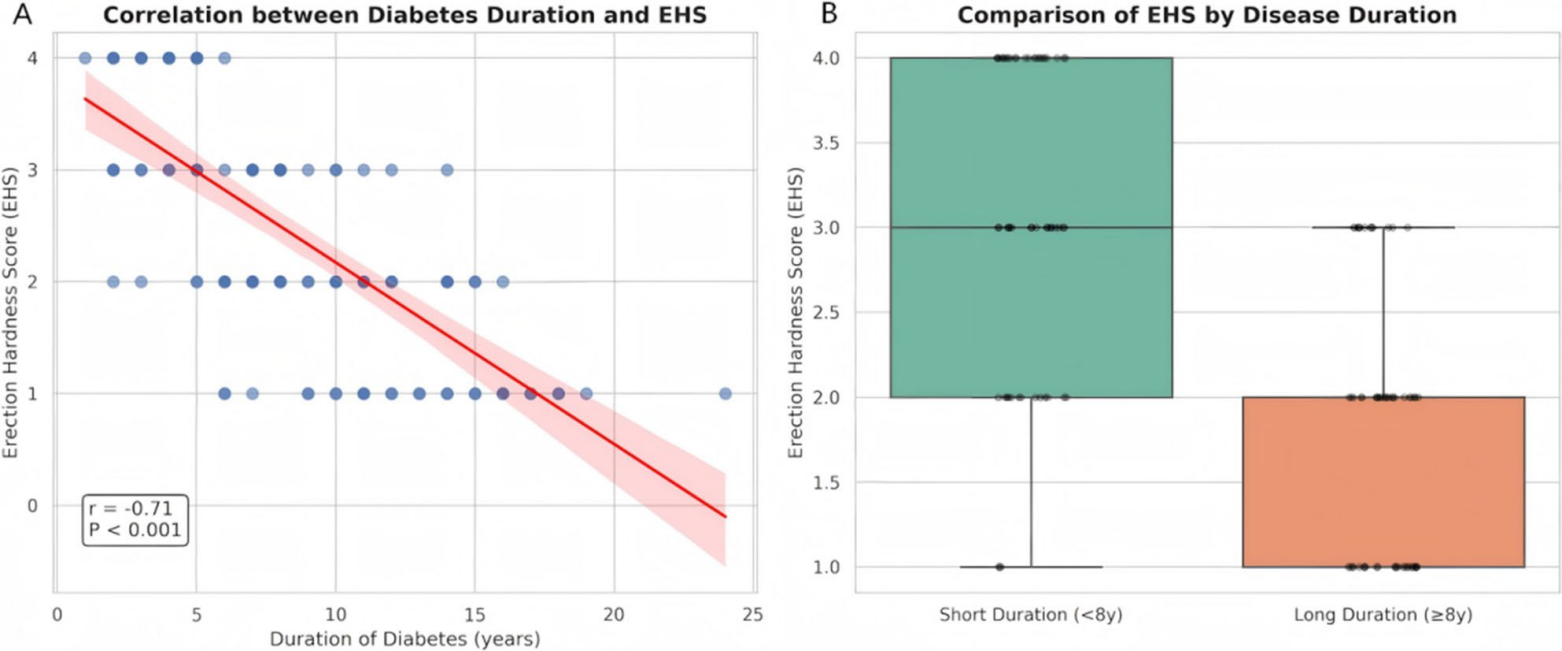
Time-Dependent Deterioration of Erectile Function in Diabetic Patients. **A** Correlation Analysis: Scatter plot with a linear regression line (red) illustrating the relationship between the duration of diabetes (in years) and the Erection Hardness Score (EHS). A strong negative correlation is observed (Pearson’s *r* = -0.71, *P* < 0.001), indicating a progressive decline in erectile rigidity as the disease duration increases. **B** Group Comparison: Box-and-whisker plot comparing the EHS distribution between patients with Short Duration (< 8 years, *n* = 56) and Long Duration (≥ 8 years, *n* = 59) of diabetes. The box represents the interquartile range (IQR), and the thick horizontal line within the box indicates the median. Individual data points are overlaid to show the spread. Patients in the Long Duration group exhibited significantly lower EHS scores compared to the Short Duration group (1.80 ± 0.76 vs. 3.07 ± 0.93, *P* < 0.001), highlighting the cumulative impact of chronic hyperglycemia on neurovascular function. *Abbreviations: EHS*,* Erection Hardness Score*

The duration of diabetes emerged as a pivotal determinant of erectile function. As illustrated in Fig. 4A, there was a strong negative correlation between diabetes duration and EHS (*r* = -0.71, *P* < 0.001). This deterioration was further confirmed by the group comparison shown in Fig. 4B. Patients in the Long Duration group (≥ 8 years) exhibited significantly lower mean EHS scores compared to those in the Short Duration group (1.80 ± 0.76 vs. 3.07 ± 0.93, *P* < 0.001). Clinically, this translated to a dramatic increase in the prevalence of moderate-to-severe ED (EHS ≤ 2), which rose from 28.6% in the short-duration group to 79.7% in the long-duration group (*P* < 0.001). Crucially, this functional decline appears to be driven specifically by the chronicity of the disease. As shown in Table 3, there were no statistically significant differences between the two groups regarding current HbA1c levels (9.63% vs. 9.98%, *P* = 0.348) or serum testosterone concentrations.

**Table 3:** Comparison stratified by disease duration

Variable	Short Duration (< 8y) (*n* = 56)	Long Duration (≥ 8y) (*n* = 59)	*P*-value
Demographics & Diabetes			
Age (years)	36.77 ± 7.69	42.83 ± 7.88	< 0.001
HbA1c (%)	9.98 ± 2.40	9.63 ± 1.48	0.348
Sexual Function			
EHS Score (Mean ± SD)	3.07 ± 0.93	1.80 ± 0.76	< 0.001
Severe ED (EHS ≤ 2), n (%)	16 (28.6%)	47 (79.7%)	< 0.001
Hormonal & Lifestyle			
Testosterone (nmol/L)	19.46 ± 3.35	18.79 ± 3.84	0.325
Smoking History, n (%)	23 (41.1%)	26 (44.1%)	0.892
Alcohol Consumption, n (%)	25 (44.6%)	16 (27.1%)	0.077

Patients were stratified by the median diabetes duration (8 years). *P*-values were calculated using independent samples t-test for continuous variables and Chi-square test for categorical variables. Statistical significance was set at *P* < 0.05

### Divergent phenotypes: type 1 vs. type 2 diabetes

A subgroup analysis stratified by diabetes type revealed a striking clinical paradox. As detailed in Table 4, patients with T1DM (*n* = 29) were significantly younger than those with T2DM (*n* = 86) (33.59 ± 8.15 vs. 42.00 ± 7.29 years, *P* < 0.001). Consistent with their younger age, T1DM patients exhibited significantly higher serum testosterone levels compared to the T2DM group (20.56 ± 3.33 vs. 18.63 ± 3.59 nmol/L, *P* = 0.013).

**Table 4:** Clinical characteristics by diabetes type

Variable	Type 1 DM (*n* = 29)	Type 2 DM (*n* = 86)	*P*-value
Demographics & History			
Age (years)	33.59 ± 8.15	42.00 ± 7.29	< 0.001
DM Duration (years)	11.55 ± 5.84	7.45 ± 3.66	< 0.001
HbA1c (%)	10.23 ± 2.39	9.66 ± 1.82	0.18
Hormonal Status			
Testosterone (nmol/L)	20.56 ± 3.33	18.63 ± 3.59	0.013
Functional Outcomes			
Moderate-to-Severe ED (EHS ≤ 2)	19 (65.5%)	44 (51.2%)	0.26
Anorgasmia (No Orgasm)	19 (65.5%)	26 (30.2%)	0.002

Comparisons between Type 1 and Type 2 Diabetes Mellitus groups. *P*<0.05 indicates statistical significance

**Fig. 5:**
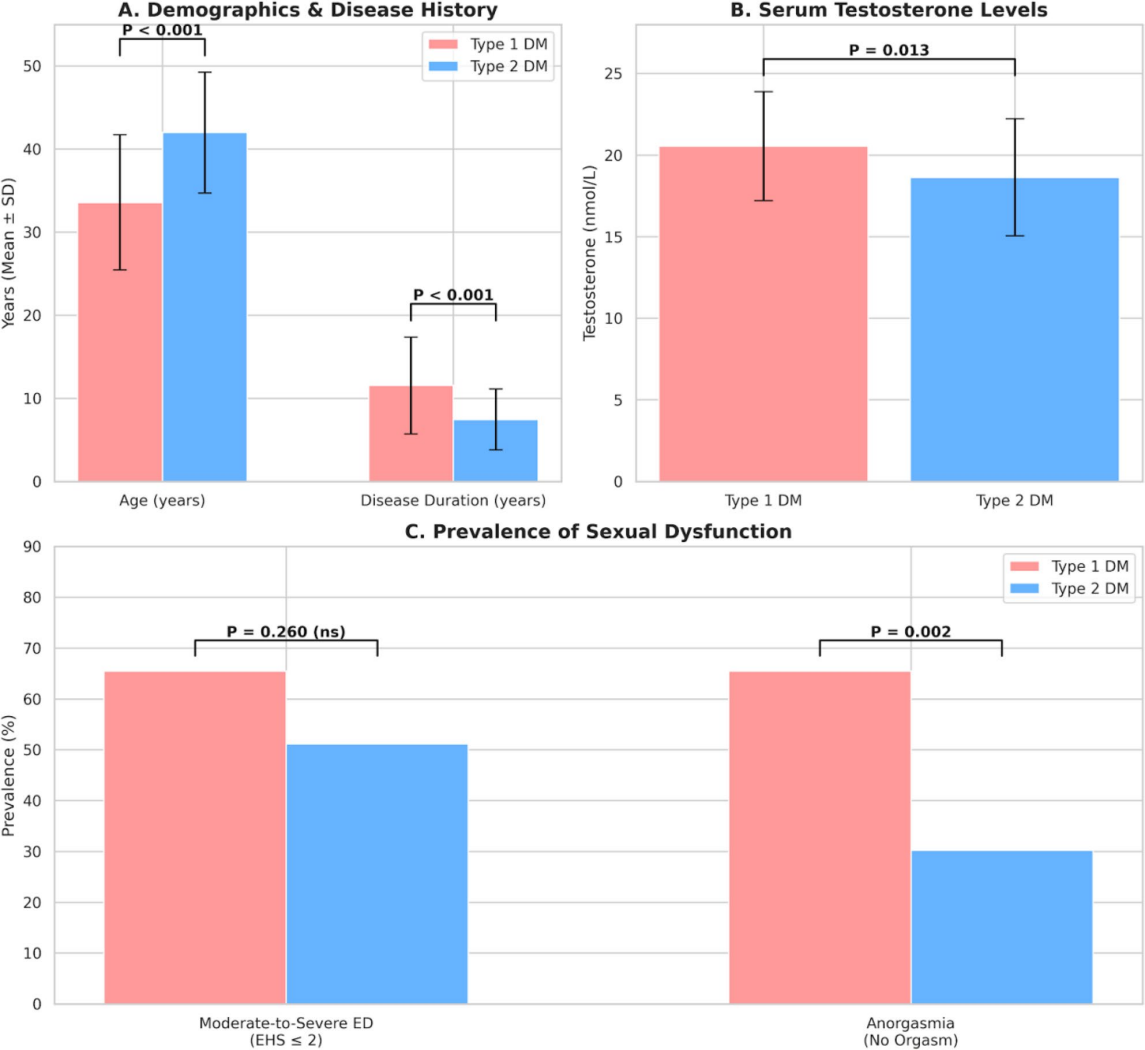
Paradoxical Discrepancy Between Clinical Profile and Functional Outcomes in Type 1 vs. Type 2 Diabetes. Comparative analysis stratified by diabetes type (Type 1, pink; Type 2, blue). **A** Demographics and Disease History: Type 1 diabetic patients were significantly younger than Type 2 patients (*P* < 0.001) yet had a significantly longer duration of disease exposure (*P* < 0.001), highlighting a “younger but chronically ill” phenotype. **B** Serum Testosterone Levels: Consistent with their younger age, Type 1 patients exhibited significantly higher mean serum testosterone levels compared to the Type 2 group (*P* = 0.013). **C** Prevalence of Sexual Dysfunction: Despite the demographic and hormonal advantages shown in A and B, Type 1 patients presented with worse functional outcomes. The prevalence of Anorgasmia (no orgasm) was more than double that of the Type 2 group (65.5% vs. 30.2%, *P* = 0.002). The rate of Moderate-to-Severe Erectile Dysfunction (EHS ≤ 2) was also higher in Type 1 patients (65.5% vs. 51.2%), though the difference was not statistically significant (*P* = 0.260). *Data are presented as Mean ± SD or percentages. P-values were calculated using Student’s t-test or Chi-square test*

However, despite these apparent advantages, T1DM patients presented with comparable, if not worse, sexual dysfunction profiles. As illustrated in Fig. 5C, the prevalence of moderate-to-severe ED (EHS ≤ 2) was surprisingly higher in the T1DM group (65.5%) compared to the T2DM group (51.2%), although this difference did not reach statistical significance (*P* = 0.260). Most notably, T1DM patients suffered from a significantly higher rate of anorgasmia. While 30.2% of T2DM patients reported anorgasmia, this rate more than doubled to 65.5% in the T1DM group (*P* = 0.002). This “younger but sicker” phenotype can likely be attributed to the significantly longer duration of disease exposure in the T1DM cohort (11.55 ± 5.84 vs. 7.45 ± 3.66 years, *P* < 0.001).

### Risk factors and independent predictors of sexual dysfunction

To comprehensively evaluate the determinants of moderate-to-severe erectile dysfunction (EHS ≤ 2), we assessed both potentially modifiable lifestyle factors and intrinsic disease characteristics through univariate comparison and multivariate logistic regression.

**Fig. 6:**
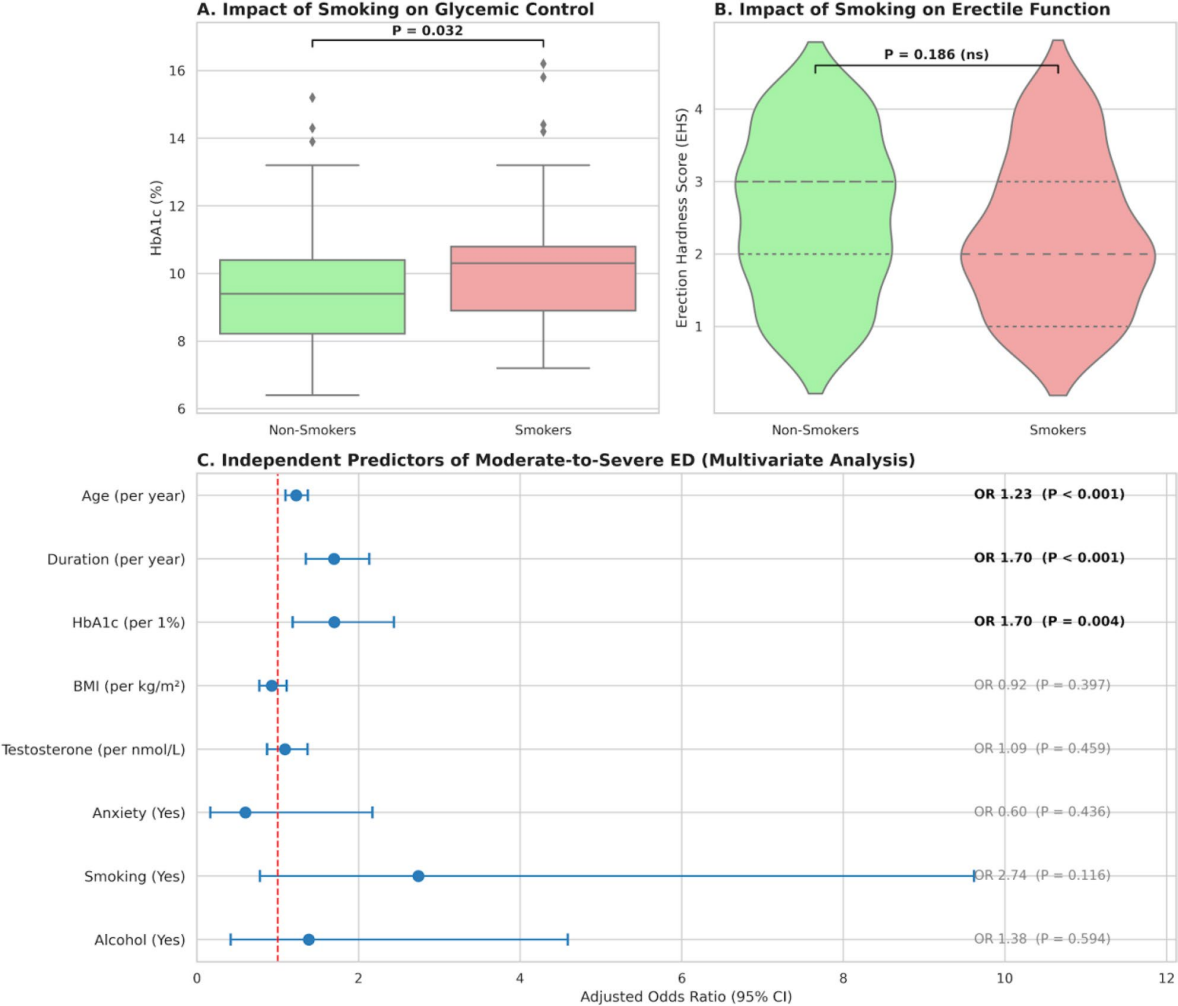
Impact of Lifestyle Factors and Independent Predictors of Moderate-to-Severe Erectile Dysfunction. **A** Impact of Smoking on Glycemic Control: Boxplot showing significantly higher HbA1c levels in smokers compared to non-smokers (*P* = 0.032), indicating a detrimental effect of smoking on metabolic stability. **B** Impact of Smoking on Erectile Function: Violin plot comparing Erection Hardness Scores (EHS) between smokers and non-smokers. Although the distribution in smokers shifts slightly lower, the difference is not statistically significant (*P* = 0.186), suggesting a “swamping” effect by other severe risk factors. **C** Multivariate Logistic Regression Analysis: Forest plot displaying the Adjusted Odds Ratios (OR) and 95% Confidence Intervals (CI) for predictors of moderate-to-severe ED. Variables to the right of the vertical red line (OR > 1) are risk factors. Age, Diabetes Duration, and HbA1c emerged as significant independent predictors (*P* < 0.05, bold text), whereas lifestyle and psychological factors were not significant in the adjusted model

Impact of Lifestyle Factors Given the high prevalence of smoking (42.6%) in the cohort, we first analyzed its impact on clinical outcomes. As shown in Fig. 6A, patients with a history of smoking exhibited significantly poorer glycemic control compared to non-smokers (HbA1c: 10.26% vs. 9.46%, *P* = 0.032). While smokers also presented with a lower mean EHS (2.27 vs. 2.53) and a higher rate of dysfunction, this difference did not reach statistical significance (*P* = 0.186, Fig. 6B), suggesting that the direct impact of smoking might be overshadowed by the severity of the underlying neuropathy. Similarly, alcohol consumption showed no significant correlation with erectile or hormonal parameters (data detailed in Table 5).

**Table 5:** Impact of lifestyle factors on clinical parameters

Variable	Non-Smokers (*n* = 66)	Smokers (*n* = 49)	*P*-value (Smoking)	Non-Drinkers (*n* = 74)	Drinkers (*n* = 41)	*P*-value (Alcohol)
Metabolic Control						
HbA1c (%)	9.46 ± 1.84	10.26 ± 2.09	0.032	10.01 ± 1.96	9.43 ± 1.98	0.129
Hormonal Status						
Testosterone (nmol/L)	19.54 ± 3.15	18.56 ± 4.12	0.15	19.32 ± 3.36	18.76 ± 4.03	0.434
Sexual Function						
EHS Score (Mean ± SD)	2.53 ± 1.07	2.27 ± 1.04	0.186	2.32 ± 1.10	2.59 ± 0.97	0.207
Moderate-to-Severe ED, n (%)	32 (48.5%)	31 (63.3%)	0.166	44 (59.5%)	19 (46.3%)	0.247

Comparisons between Smokers vs. Non-Smokers, and Drinkers vs. Non-Drinkers. *P*-values derived from t-tests (for HbA1c, Testosterone, EHS) or Chi-square tests (for ED prevalence)

Multivariate Logistic Regression Analysis To isolate the independent predictors of severe dysfunction, a multivariate model was constructed incorporating age, diabetes duration, HbA1c, hormonal status, and lifestyle factors. The results are visualized in the forest plot in Fig. 6C and detailed in Table 6.

**Table 6:** Logistic regression analysis

Variable	Univariate OR (95% CI)	*P*-value	Multivariate OR (95% CI)	*P*-value
Age (per year)	1.16 (1.08–1.23)	< 0.001	1.23 (1.10–1.37)	< 0.001
Duration (per year)	1.55 (1.32–1.82)	< 0.001	1.70 (1.35–2.13)	< 0.001
HbA1c (per 1%)	1.09 (0.90–1.32)	0.37	1.70 (1.18–2.44)	0.004
BMI (per kg/m²)	0.97 (0.89–1.07)	0.607	0.92 (0.77–1.11)	0.397
Testosterone (per nmol/L)	0.92 (0.83–1.02)	0.132	1.09 (0.87–1.37)	0.459
Anxiety/Depression (Yes)	2.24 (1.03–4.88)	0.042	0.60 (0.17–2.17)	0.436
Smoking (Yes)	1.83 (0.86–3.89)	0.117	2.74 (0.78–9.62)	0.116
Alcohol (Yes)	0.59 (0.27–1.27)	0.177	1.38 (0.42–4.59)	0.594

Univariate and multivariate logistic regression models assessing risk factors for Moderate-to-Severe Erectile Dysfunction (EHS ≤ 2). Adjusted Odds Ratios (OR) account for age, diabetes duration, HbA1c, hormonal status, and lifestyle factors. CI denotes Confidence Interval

After adjusting for confounders, three key variables emerged as significant independent risk factors:1.Diabetes Duration: This remained the strongest predictor. Each additional year of diabetes increased the risk of severe ED by 70% (Adjusted OR 1.70, *P* < 0.001)0.2.Age: Increasing age was confirmed as an independent risk factor (Adjusted OR 1.23, *P* < 0.001)0.3.Glycemic Control (HbA1c): Crucially, multivariate analysis unmasked the true impact of hyperglycemia. While not significant in univariate analysis, HbA1c became a strong independent predictor after adjusting for duration and age (Adjusted OR 1.70, *P* = 0.004).

In contrast, lifestyle factors (smoking, alcohol) and psychological comorbidities lost statistical significance in the adjusted model. This indicates that in this population with advanced complications, the fundamental characteristics of diabetes (chronicity and metabolic instability) are the primary drivers of sexual failure.

## Discussion

### Overview of main findings

This retrospective study provides a comprehensive clinical profile of 115 diabetic men with RE. Our findings challenge the traditional view of RE as an isolated fertility issue. Instead, we identified a pervasive “triple dysfunction” syndrome characterized by the co-occurrence of RE, moderate-to-severe ED, and anorgasmia. As noted by Mostafa and Abdel-Hamid [12], EjD in diabetes is often the “forgotten” complication, overshadowed by ED, yet our data suggests they are inextricably linked manifestations of the same systemic pathology.

### The “Triple Dysfunction” syndrome: neurogenic and psychological mechanisms

The high prevalence of co-morbid **E**D (80.0%) and anorgasmia (39.1%) in our cohort underscores the systemic nature of diabetic autonomic neuropathy. Ejaculation is a complex reflex arc coordinated by the spinal ejaculation generator. As elucidated by Qiu et al. [4], autonomic nervous system disorders disrupt the sympathetic innervation of the bladder neck, leading to closure failure. Furthermore, Chéhensse et al. [13] have mapped the spinal generator located in the lumbar-sacral spinal cord, showing that its dysregulation affects both emission and expulsion. Our findings confirm that when this neural circuitry is compromised enough to cause RE, it almost invariably signals concurrent damage to the cavernosal nerves responsible for erection. This aligns with Peng et al. [14], who reported that in Chinese diabetic patients, ED is characterized by more severe organic damage compared to non-diabetic ED, necessitating early and aggressive intervention.

### Psychological burden and management gaps

The burden of this dysfunction extends beyond physiology. We observed a significant association between anxiety/depression and severe ED scores. This is consistent with the recent findings of Rathod et al. [9], who emphasized that sexual dysfunction in diabetic males significantly impairs marital adjustment and exacerbates depressive symptoms, creating a vicious cycle of psychogenic and organic failure. Furthermore, Kang et al. [15] highlighted a critical gap in diabetes management across Asia, noting that men’s sexual health is frequently overlooked in routine metabolic care. Our study reinforces the need for a holistic approach; clinicians treating RE must screen for occult psychological distress to break this cycle.

### The type 1 diabetes paradox: younger age, greater damage

A striking finding of our study was the “T1DM Paradox”: T1DM patients, despite being significantly younger and having higher testosterone levels, exhibited worse functional outcomes. This corroborates Hylmarova et al. [2], who noted that T1DM men often develop sexual dysfunction earlier in life due to the longer duration of insulin dependency and early-onset microvascular damage. Our data confirms that disease duration is the true “biological clock” of sexual decline. The preservation of testosterone levels suggests that androgen replacement therapy may have limited efficacy in this subgroup, where neurogenic damage predominates.

### Metabolic control and systemic toxicity

Our multivariate analysis identified HbA1c as a potent independent predictor of severe dysfunction, highlighting the systemic toxicity of hyperglycemia. Rong et al. [5] recently elucidated the multilayered mechanisms by which diabetes impairs reproductive function, emphasizing that oxidative stress and inflammatory pathways contribute to irreversible damage in both spermatogenesis and the neurovascular bed. Additionally, Boeri et al. [16] have shown that sexual dysfunction can begin even in the prediabetic stage, suggesting that metabolic insults accumulate long before the clinical diagnosis of RE.

### Implications for fertility management

Given that RE is a major cause of male infertility [17], accurate diagnosis and management are critical. Our study confirms that while spontaneous fertility is compromised, the potential for assisted reproduction remains. Álvarez et al. [7] recently demonstrated that optimized protocols for sperm recovery from urine can yield viable sperm in men with RE. Similarly, Perrin et al. [6], in a study published in *Basic and Clinical Andrology*, showed that using modified retrieval techniques can lead to successful pregnancies and healthy live births. These findings collectively advocate for early referral to reproductive specialists for diabetic men presenting with low ejaculate volume.

### Limitations

Several limitations of this study must be acknowledged. First, the retrospective design limits our ability to establish causality between risk factors and the progression of dysfunction. Second, the sample size was relatively small and recruited from a single center, which may limit the generalizability of the findings to broader diabetic populations. Third, while we used validated scales for ED, the assessment of orgasm and psychological status relied partly on self-reporting, which introduces potential recall bias. Finally, we did not perform advanced neurophysiological testing (e.g., bulbocavernosus reflex latency) to objectively quantify the extent of neuropathy. Future prospective, multi-center studies with larger cohorts and objective neuro-testing are warranted to validate these findings.

## Conclusion

In conclusion, our study reveals distinct patterns of sexual dysfunction between Type 1 and Type 2 diabetes mellitus patients with retrograde ejaculation. While T1DM patients present with better hormonal profiles, they paradoxically exhibit poorer erectile function, largely driven by a longer disease duration. Our findings identify disease duration as the most significant independent predictor for moderate-to-severe ED, outweighing the impact of diabetes type or age alone. These results underscore the critical need for early screening and individualized management of sexual dysfunction in diabetic men, particularly for those with a long-standing history of the disease.

## Data Availability

The data and materials used in this study are available from the correspondingauthor upon reasonable request.

## Abbreviations

Abbreviation: Full Term
BMI: Body Mass Index
CI: Confidence Interval
DM: Diabetes Mellitus
ED: Erectile Dysfunction
EHS: Erection Hardness Score
EjD: Ejaculatory Dysfunction
HbA1c: Glycated Hemoglobin
HPF: High Power Field
IQR: Interquartile Range
OR: Odds Ratio
RE: Retrograde Ejaculation
SD: Standard Deviation
T1DM: Type 1 Diabetes Mellitus
T2DM: Type 2 Diabetes Mellitus
WHO: World Health Organization
